# Correlation of patient characteristics with arm and finger measurements in Asian parturients: a preliminary study

**DOI:** 10.1186/s12871-020-01131-6

**Published:** 2020-08-31

**Authors:** Ming Jian Lim, Chin Wen Tan, Hon Sen Tan, Rehena Sultana, Victoria Eley, Ban Leong Sng

**Affiliations:** 1 Department of Women’s Anesthesia, KK Women’s and Children’s Hospital, 100 Bukit Timah Road, Singapore, 229899 Singapore; 2 Duke-NUS Medical School, 100 Bukit Timah Road, Singapore, 229899 Singapore; 3 Centre for Quantitative Medicine, Duke-NUS Medical School, Singapore, Singapore; 4 Department of Anesthesia and Perioperative Medicine, The Royal Brisbane and Women’s Hospital, Brisbane, Australia

**Keywords:** Body weight, Blood pressure, Obstetrics

## Abstract

**Background:**

Accurate blood pressure (BP) measurement depends on appropriate cuff size and shape in relation to the arm. Arm dimensions outside the recommended range of cuff sizes or trunco-conical arms may result in inaccurate BP measurements. Measuring BP using finger cuffs is a potential solution. Arm cuff size is based on mid-arm circumference (MAC), and trunco-conicity is quantified by conicity index. We aimed to determine the correlation of MAC, body mass index (BMI), and weight with conicity index.

**Methods:**

A prospective cohort study was conducted in the KK Women’s and Children’s Hospital where third trimester parturients scheduled for cesarean delivery were recruited after obtaining informed consent. Parturients were asked to rate their experience with time taken to obtain BP readings, cuff popping off during measurement, need to move the cuff from the upper arm to lower arm or leg, and need to change to a different cuff. Our primary outcome was the correlation between MAC and conicity index, calculated using Pearson’s correlation. The correlation between BMI and weight with conicity index was also determined.

**Results:**

We enrolled 300 parturients. Moderate correlation was found between left MAC and left conicity index (r = 0.41, 95% CI 0.32 to 0.51), and right MAC and right conicity index (r = 0.39, 95% CI 0.29 to 0.48). Weight (r = 0.35 to 0.39) and BMI (r = 0.41 to 0.43) correlated with conicity index in this study. MAC of 1 parturient fell outside the recommended range for arm cuffs, but all parturients fit into available finger cuffs. Obese parturients had increased problems with arm cuffs popping off and needing a change of cuff.

**Conclusions:**

BMI better correlated with conicity index compared to MAC or weight. Standard finger cuffs were suitable for all parturients studied and may be a suitable alternative.

**Trial registration:**

Clinicaltrials.gov NCT04012151. Registered 9 Jul 2019

## Background

Accurate measurement of blood pressure (BP) is important in peripartum care [1]. Mercury sphygmomanometers or hybrid devices with cylindrical arm cuffs remain the gold standard for BP measurement [2], however, poorly fitting arm cuffs can yield inaccurate BP readings, potentially leading to inappropriate clinical management [3].

Accurate BP measurements require the cuff to fit the parturient’s arm in two respects; appropriate cuff size in relation to the parturient’s arm circumference, and a cuff shape that matches that of the parturient’s arm [4]. Cuff size is often based on the mid-arm circumference (MAC), defined as the circumference at the mid-point of the upper arm, with appropriately-sized cuff bladder dimensions being 40% width and 80% length of the MAC, respectively [5–7]. However, sizing cuffs using these criteria overestimate BP in patients with small MAC, while underestimating BP in patients with higher MAC [8]. This issue may be magnified in obese parturients, given that appropriately sized cuffs are not always readily available. A study conducted on 450 Australian parturients reported that 12.9 and 1.3% of parturients requiring “large” and “thigh” cuffs, respectively, while none of the available cuff sizes produced an acceptable fit in 3.1% of parturients and required cuff placement on the forearm or leg [3]. The erroneous use of inappropriately small cuffs in obese parturients overestimates BP [9, 10], with potential risk for significant adverse outcomes [11].

Additionally, the shape of the cuff should match that of the arm. In most individuals, the shape of the upper arm has been described as trunco-conical (truncated cone) instead of cylindrical [4]. The degree of trunco-conicity varies depending on gender and obesity [4], and can be mathematically described using a conicity index derived from arm length, proximal and distal arm circumferences [12]. In parturients with higher conicity indices, the large gap between the BP cuff and the surface of the distal arm [9, 12] results in irregular expansion of the cylindrical BP cuff during inflation and overestimation of systolic BP by up to 9.7 mmHg and diastolic BP by up to 7.8 mmHg [13]. Again, this issue may be exacerbated in obese patients, as BMI is moderately correlated with arm conicity index (r = 0.51, r^2^ = 0.26) [3].

A potential solution to inappropriately sized cuffs or trunco-conical arms may lie with the use of finger cuff devices such as the Nexfin™ monitor (BMEYE B.V., Holland), CNAP™ monitor (CNSystems Medizintechnik AG, Graz, Austria), and Clearsight™ (Edwards Lifesciences, Irvine, California, USA). In comparison to arm cuffs, finger cuffs from Clearsight™ accommodated up to finger circumference of 6.8 cm and failed to fit only 0.7% of Australian parturients with maximum finger circumference of 7 cm [3]. In contrast, CNAP™ finger cuff device accommodates finger circumferences up to 8.8 cm and would fit all parturients in the study cohort.

Although the issue of inappropriate cuff size or shape for BP measurement have been established in the Caucasian population, it is yet unclear if this is applicable to Asian parturients. The MAC in Asian parturients have not been determined; a study by Wang et al. found no differences in MAC of non-pregnant Caucasians and Asians [14], but it is uncertain if the available range of arm cuffs will fit Asian parturients. Second, because arm conicity index measurements and its association with BMI, MAC, and weight have not been established in Asian parturients, we are uncertain if the issue of trunco-conicity applies to Asian parturients and how it may vary according to parturient characteristics. Finally, the suitability of available finger cuff sizes in Asian parturients has not been established. Hence, this prospective cohort study aimed to determine MAC within our Asian parturient population and its correlation with conicity index. Secondarily, we investigated the association between BMI and weight with arm conicity index, as well as the ability of finger cuffs from Nexfin™ and CNAP™ to fit the finger circumferences of Asian parturients.

## Methods

This study adheres to the applicable Strengthening the Reporting of Observational studies in Epidemiology (STROBE) guidelines [15]. Informed consent was obtained from all parturients, and ethics approval was obtained from the SingHealth Centralized Institutional Review Board (Reference number: CIRB 2019/2290) and registered on Clinicaltrial.gov (ID: NCT04012151).

This was a prospective cohort study in which parturients scheduled for elective cesarean delivery were enrolled at the pre-anesthetic clinic in KK Women’s and Children’s Hospital (KKH) from July to December 2019. Consecutive eligible parturients were enrolled. Eligible parturients were between 21 to 50 years old, at 32 or more weeks of gestation, and American Society of Anesthesiology (ASA) Physical Status I to III. Parturients undergoing unscheduled cesarean delivery were excluded.

Information on baseline demographic and obstetric characteristics were obtained from the patients or their medical records on admission for surgery. BMI was calculated from the parturient’s height and weight at enrollment. Measurements from both upper limbs were taken according to standard anthropometry protocols when available [16]. Arm length was measured from the tip of the acromion process to the tip of the olecranon process on the posterior aspect of the arm with the elbow flexed [16]. With the arm hanging by the side, MAC was measured at the mid-point of the arm length [16], the proximal arm circumference was measured at the axilla, and the distal arm circumference was measured at the elbow above the elbow crease. Finger circumference was measured at the mid-point of the middle phalanx of the middle finger, with the hand placed flat on a table. As described by Bonso et al. [12], arm conicity index was calculated as:

$$ \frac{\mathrm{Proximal}\ \mathrm{arm}\ \mathrm{diameter}-\mathrm{Distal}\ \mathrm{arm}\ \mathrm{diameter}}{\mathrm{Arm}\ \mathrm{length}}\ \mathrm{X}\ 100 $$

Arm diameter was calculated by dividing the relevant arm measurement by π. The appropriate arm or finger cuff size was determined based on MAC and finger circumference respectively, in accordance with manufacturers’ recommendations. Patients also responded to a three-point rating scale (never/sometimes/always) about their experience of the procedure for BP measurements during the current pregnancy, with questions asking about extended waiting period to obtain a reading, whether the cuff pops off during measurement, the need to take BP on the lower arm or the leg, and whether they needed to change to a different cuff.

### Sample size calculation and statistical analysis

A sample of 300 parturients was adequate to estimate a Pearson’s correlation coefficient of at least 0.4 with a 95% CI width of 0.2, based on the correlation results reported by a previous study methodology on a Caucasian population [3].

Our primary outcome was the correlation between MAC and conicity index, which was reported using Pearson’s correlation with a 95% confidence interval (CI) for each arm. Univariate linear regression was used to analyze the quantitative association between conicity index and MAC, BMI, and weight. Association between conicity index and other variables were expressed as β estimate with its 95% CI. Parturient characteristics, arm anthropometric data and patient’s experience on BP measurement was summarized according to obesity status (BMI < 30 kg/m^2^ as non-obese; BMI ≥30 kg/m^2^ as obese). Categorical and continuous variables were summarized as frequency (proportions) and as mean (standard deviation (SD), minimum - maximum). Univariate logistic regression model was fitted to determine associations between each of patient’s experience survey questions with obesity status. Association from logistic regression analysis was expressed as odds ratio (OR) with 95% CI. A statistical significance was set at *p*-value < 0.05. All the analysis was done using SAS 9.4 software.

## Results

We screened 377 parturients, of whom 77 refused consent. Hence, 300 parturients were enrolled, with their characteristics and gestational information shown in Table 1. Of the 300 parturients, 194 (64.7%) were non-obese while 106 (35.3%) were obese. The mean (SD) BMI in non-obese and obese group parturients was 26.2 (2.5) kg/m^2^ and 34.5 (4.0) kg/m^2^ respectively. Arm and finger measurements, along with conicity indices are summarized in Table 2.

**Table 1:** Parturient and gestational characteristics

	Non-obese^a^ ***N*** = 194	Obese^a^ ***N*** = 106	Total ***N*** = 300
Age (years)	33.9 ± 4.4	33.7 ± 3.7	33.8 ± 4.2
Gestational age (weeks)	38.2 ± 1.1	38.1 ± 1.0	38.2 ± 1.1
Height (m)	1.6 ± 0.1	1.6 ± 0.1	1.6 ± 0.1
Weight (kg)	66.1 ± 8.0	86.5 ± 11.4	73.3 ± 13.5
BMI (kg/m^2^)	26.2 ± 2.5	34.5 ± 4.0	29.1 ± 5.1
Race / ethnicity			
Chinese	119 (61.3)	48 (45.3)	167 (55.7)
Malay	36 (18.6)	34 (32.1)	70 (23.3)
Indian	15 (7.7)	14 (13.2)	29 (9.7)
Others	24 (12.4)	10 (9.4)	34 (11.3)
ASA physical status			
I	98 (50.5)	32 (30.2)	130 (43.3)
II	95 (49.0)	62 (58.5)	157 (52.3)
III	1 (0.5)	12 (11.3)	13 (4.3)
Chronic hypertension	3 (1.5)	2 (1.9)	5 (1.7)
Gestational hypertension^b^	6 (3.1)	5 (4.7)	11 (3.7)
Gestational diabetes	18 (9.3)	14 (13.2)	32 (10.7)

Values are represented as mean ± standard deviation (SD) or number (%)

*ASA* American Society of Anesthesiologists, *BMI* body mass index

^a^ Obese and non-obese groups were defined as BMI ≥ 30 kg/m^2^ and BMI < 30 kg/m^2^ respectively

^b^ Gestational hypertension defined as having a blood pressure ≥ 140/90 on two separate occasions more than 6 h apart, without proteinuria and diagnosed after 20 weeks of gestation

**Table 2:** Arm and finger measurements

	Non-obese^a^ N = 194	Obese^a^ N = 106	Total N = 300
Right arm measurements (cm)			
Length	33.9 ± 2.2 [28, 40]	34.4 ± 2.5 [26, 41]	34.1 ± 2.3 [26, 41]
Proximal circumference	29.1 ± 3.3 [20, 38]	35.0 ± 5.0 [14, 49]	31.4 ± 4.9 [14, 49]
MAC	25.5 ± 2.6 [20, 36]	31.4 ± 4.0 [15, 43]	27.6 ± 4.2 [15, 43]
Distal circumference	23.7 ± 2.3 [19, 34]	28.7 ± 3.6 [13, 40]	25.4 ± 3.7 [13, 40]
Left arm measurements (cm)			
Length	33.9 ± 2.2 [27, 41]	34.4 ± 2.4 [26, 40]	34.1 ± 2.3 [26, 41]
Proximal circumference	29.1 ± 3.3 [19, 38]	35.5 ± 4.5 [14, 49]	31.2 ± 4.9 [14, 49]
MAC	25.5 ± 2.7 [19, 37]	31.4 ± 4.0 [15, 43]	27.6 ± 4.3 [15, 43]
Distal circumference	23.7 ± 2.4 [19, 35]	28.7 ± 3.6 [13, 41]	25.5 ± 3.7 [13, 41]
Right arm conicity index (%)	5.1 ± 2.0 [1, 12]	6.4 ± 2.3 [1, 14]	5.6 ± 2.2 [1, 14]
Left arm conicity index (%)	4.8 ± 1.9 [0, 10]	6.2 ± 2.0 [1, 12]	5.3 ± 2.1 [0, 12]
Right finger circumference (cm)	5.2 ± 0.3 [5, 6]	5.5 ± 0.4 [5, 7]	5.3 ± 0.4 [4, 7]
Left finger circumference (cm)	5.1 ± 0.3 [4, 6]	5.5 ± 0.3 [5, 7]	5.2 ± 0.4 [4, 7]

Values are represented as mean ± standard deviation (SD) [min, max]

*BMI* body mass index; *MAC* mid-arm circumference

^a^ Obese and non-obese groups were defined as BMI ≥ 30 kg/m^2^ and BMI < 30 kg/m^2^ respectively

### Correlation between MAC and arm conicity indices

Our primary objective was to determine the correlation between MAC and arm conicity indices. The correlation between the left MAC and left arm conicity index (r = 0.41, 95% CI 0.32–0.51) was similar to the right MAC and right arm conicity index (r = 0.39, 95% CI 0.29–0.48).

### Association between BMI, weight, and MAC with conicity indices

We found a moderate correlation between MAC, weight, and BMI with their respective conicity indices (Table 3). Of the three variables, BMI correlated the best with conicity index for both arms (left: r = 0.43; right: r = 0.41). Due to significant collinearity between MAC, weight, and BMI (collinearity indices > 0.8), three separate univariate linear regression models were performed, which showed significant associations between BMI, weight, and MAC with the respective conicity indices. Each unit increase in BMI increased both arm conicity indices by 0.18.

**Table 3:** Univariate linear regression analysis describing the associations between BMI, weight, and MAC with conicity indices

	ß (95% CI)	r (95% CI) ^a^	***p***-value
Right conicity index			
MAC	0.20 (0.25–0.26)	0.39 (0.29–0.48)	< 0.01
Weight	0.06 (0.04–0.07)	0.35 (0.25–0.45)	< 0.01
BMI	0.18 (0.13–0.22)	0.41 (0.31–0.50)	< 0.01
Left conicity index			
MAC	0.20 (0.15–0.25)	0.41 (0.32–0.51)	< 0.01
Weight	0.06 (0.04–0.07)	0.39 (0.29–0.48)	< 0.01
BMI	0.18 (0.13–0.22)	0.43 (0.34–0.52)	< 0.01

*BMI* body mass index, *CI* confidence interval, *MAC* mid-arm circumference

^a^ Pearson correlation coefficient

### Distribution of recommended arm and finger cuff sizes according to MAC

**Fig. 1:**
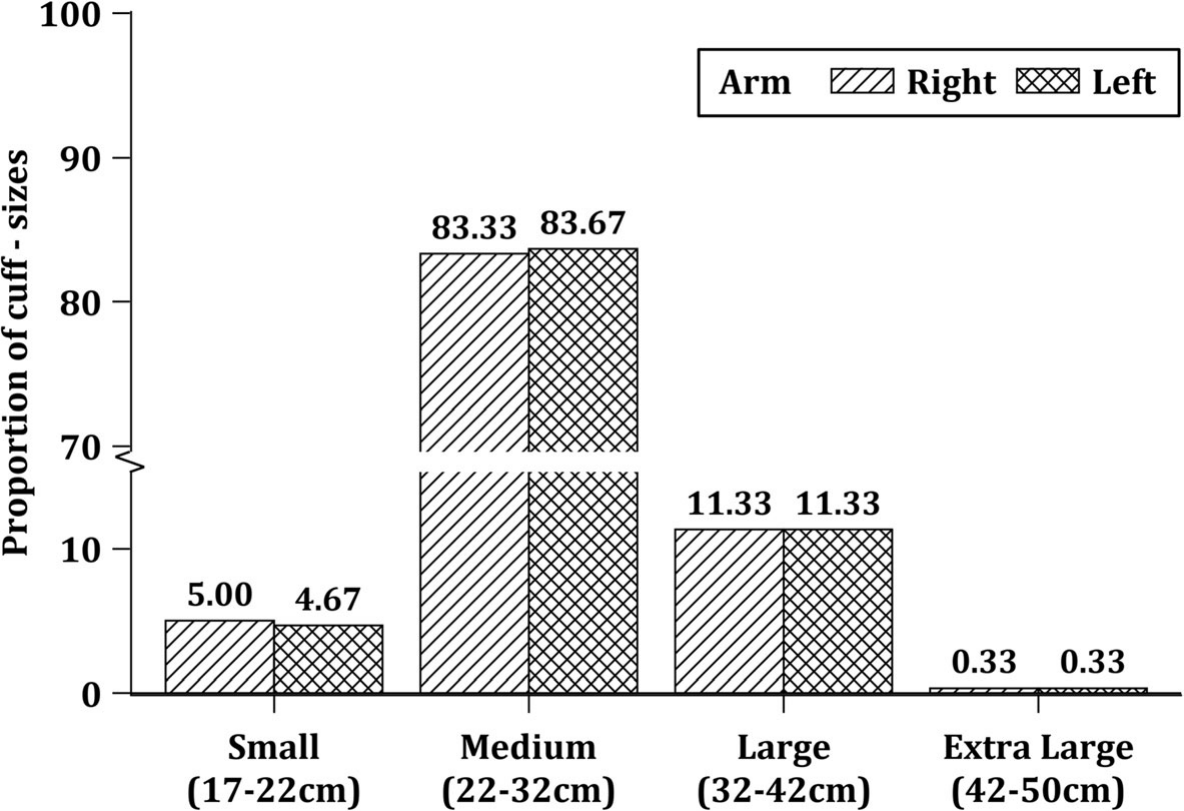
Frequency distribution of left and right arm cuff sizes (*n* = 300). Data presented as % of study population

**Fig. 2:**
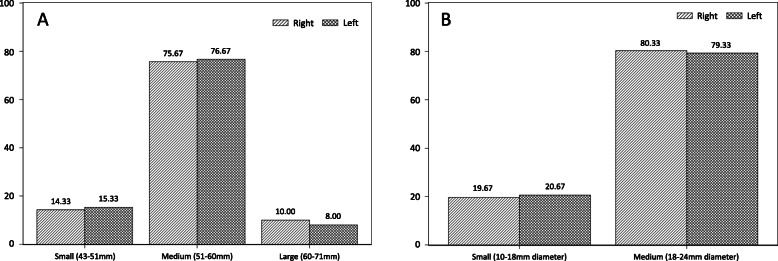
Frequency distribution of left and right finger cuff sizes for Nexfin™ or CNAP™ devices (n = 300). Data presented as % of study population

Based on MAC, the frequency distribution of arm cuff sizes according to American Heart Association (AHA) recommendations are illustrated in Fig. 1. One parturient had left and right arm circumferences of less than 17 cm, which was below the recommended range of the smallest arm cuff. None of the parturients had MAC above the range of the largest arm cuff. Similarly, fitting of the Nexfin™ or CNAP™ finger cuffs were based on finger circumferences as recommended by the manufacturer (Fig. 2). One parturient had a left finger circumference that was less than 43 mm, which was below lower range of the smallest Nexfin finger cuff. However, this parturient’s right finger circumference was 45 mm, which fell within the recommended finger cuff range. In contrast, none of the parturients’ finger circumferences fell outside the recommended measurements for CNAP™.

### Parturient experience survey

Obese parturients were more likely to experience the arm cuff popping off (OR 10.18, 95% CI 4.04–25.69) or needing a cuff change (OR 9.72, 95% CI 3.19–29.56), compared to non-obese parturients. In addition, the parturient experience survey found that 28 (9.3%) parturients experienced an extended waiting period while taking a BP measurement, and 4 (1.3%) parturients required the cuff to be put on the lower arm or leg, of whom 3 (2.8%) were obese (Table 4).

**Table 4:** Parturient satisfaction survey regarding blood pressure measurements

Question	Non-obese^a^ (N = 194)	Obese^a^ (N = 106)	Unadjusted odds ratio (95% CI)	***p***-value
Long time taken for blood pressure reading	19 (9.8)	9 (8.5)	0.86 (0.37–1.96)	0.71
Cuff pops off	6 (3.1)	26 (24.5)	10.18 (4.04–25.69)	< 0.01
Need to put cuff on lower arm or leg	1 (0.5)	3 (2.8)	5.62 (0.58–54.70)	0.14
Need to change to different cuff	4 (2.1)	18 (17.0)	9.72 (3.19–29.56)	< 0.01

Values are represented as number (%)

*BMI* body mass index, *CI* confidence interval

^a^ Obese and non-obese groups were defined as BMI ≥ 30 kg/m^2^ and BMI < 30 kg/m^2^ respectively

## Discussion

Our results showed moderate correlation between MAC and arm conicity index in third trimester parturients. Furthermore, although MAC, weight, and BMI showed moderate correlation with arm conicity, BMI had the highest correlation coefficient with arm conicity, with every unit increase in BMI increasing the conicity index by 0.18. Out of 300 parturients, one had MAC below the recommended range for arm cuffs. However, all parturients were able to fit into one of the standard Nexfin™ and CNAP™ finger cuffs. Compared to non-obese parturients, obese parturients experienced significantly more problems with BP measurement, including arm cuffs popping off, or requiring a change in BP cuffs.

Our finding that MAC is moderately correlated with conicity index is consistent with other studies of Caucasian parturients, suggesting that this correlation applies to our multi-ethnic Asian population. Bonso et al. were the first to mathematically quantify arm conicity using a conicity index [12], and despite using non-standard anthropometry techniques and arm length measurements, Bonso et al. and Palatini et al. demonstrated that MAC was correlated with arm conicity index [12, 13]. Subsequently, Eley et al. increased the robustness and reproducibility of arm length measurements by utilizing bony landmarks based on the Anthropometry Procedures Manual of the Center for Disease Control [3, 16], and reported a similar correlation between MAC and conicity index [3].

In addition to MAC, BMI and weight were also correlated with conicity index, and of the three variables, BMI was found to correlate the best with arm conicity. These results are similar to that of Eley et al., who also reported that BMI correlated best with arm conicity, accounting for 26% of the variation in conicity index [3]. Furthermore, our finding that BMI, weight, and MAC were collinear was also reported by Eley et al. [3]. This may be explained by the association between increasing BMI and higher MAC in both pregnant [3, 17, 18] and non-pregnant populations [19], and is likely due to an increase in arm fat mass as measured using bioelectrical impedance analysis [17]. Additionally, obese parturients were more likely to experience challenges during BP measurement, with an increased incidence of the arm cuffs popping off and the need to change to a different cuff, compared to non-obese parturients. These findings were echoed by Eley et al. [3].

Our findings that obesity is associated with increasing MAC and arm conicity is concerning. Although the highest MAC measured in our study did not exceed the recommended MAC range of the largest cuff, these non-standard cuff sizes may not be readily available, and shifting the point of measurement from the upper arm to lower arm is unacceptable as it has been shown to overestimate BP [20–22]. Furthermore, the increase in conicity index may cause irregular expansion of the cylindrical cuff during inflation, leading to overestimation of both systolic and diastolic BP [13]. Although the use of cone-shaped BP cuffs may improve the accuracy of BP measurements compared to cylindrical cuffs [23], the former is not available at our institution. Nonetheless, the increased risk of overestimating BP in obese parturients is clinically significant given that obese parturients are already at elevated baseline risk for hypertensive disorders [2, 13], with the prevalence of obesity in Singapore likely to increase further [24].

The potential solution to the increased MAC and conicity index of obese parturients may lie with the use of finger cuff devices like the Nexfin™ and CNAP™. Our results demonstrated that all parturients were able to fit into the standard finger cuffs provided by Nexfin™ and CNAP™, which may provide an alternative means of measuring BP in parturients who do not fit the available arm cuffs. Nexfin™ has been validated against sphygmomanometry in pregnant patients, and passed both Association for the Advancement of Medical Instrumentation (AAMI) standards and European Society of Hypertension International Protocol Phases 1 and 2.1 [25]. Similarly, CNAP™ has been validated against intra-arterial BP in general surgery patients under anesthesia and met AAMI standards [26]. In contrast, the accuracy of finger cuff-based BP measurements were unacceptable by AAMI standards in critically ill patients receiving vasopressor infusions or with finger edema [27], and should not be used as an alternative to arm cuffs in this patient population. However, our standard practice for such patients is intra-arterial BP monitoring.

We acknowledge several limitations to this study. Since BP measurements using arm or finger cuff-based systems were not recorded and compared against another standard such as intra-arterial BP, we are unable to assess the implications of increasing MAC or conicity index on BP measurement accuracy, and this should be determined in future studies. This information will help determine if finger cuff-based BP measurements should be used in parturients with high arm conicity or who do not fit standard arm cuffs. In addition, we enrolled parturients who were scheduled for elective cesarean delivery, which may not be representative of the general obstetric population since parturients with higher BMI are more likely to undergo cesarean delivery [3, 28]. Finally, we enrolled parturients in their third trimester, raising concerns that gestational weight gain during the interval prior to cesarean delivery will lead to increased BMI and MAC. However, Hogan et al. studied MAC in parturients across all three trimesters and found that the mean MAC did not vary significantly with different trimesters of pregnancy [17].

## Conclusions

In summary, our study reported that BMI is better correlated with arm conicity index, compared to MAC or weight. Obese parturients are at increased risk of having issues during BP measurement, such as cuffs popping off or requiring a change of cuff. Validated finger cuff devices such as the Nexfin™ and CNAP™ may be an alternative means for BP measurement in patients who are unsuited to traditional arm cuffs. Further studies are required to assess the implications of increasing MAC and arm conicity on BP measurement accuracy.

## Data Availability

The datasets generated and analyzed in this work are available for anyone who wishes to access the data by contacting the corresponding author.

## Abbreviations

AAMI: Association for the Advancement of Medical Instrumentation
AHA: American Heart Association
ASA: American Society of Anesthesiologists
BMI: Body mass index
BP: Blood pressure
CI: Confidence interval
MAC: Mid-arm circumference
OR: Odds ratio
SD: Standard deviation
STROBE: Strengthening the Reporting of Observational studies in Epidemiology
